# The risk of stroke/systemic embolism and major bleeding in Asian patients with non-valvular atrial fibrillation treated with non-vitamin K oral anticoagulants compared to warfarin: Results from a real-world data analysis

**DOI:** 10.1371/journal.pone.0242922

**Published:** 2020-11-30

**Authors:** Oh Young Bang, Young Keun On, Myung-Yong Lee, Sung-Won Jang, Seongwook Han, Sola Han, Mi-Mi Won, Yoo-Jung Park, Ji-Min Lee, Hee-Youn Choi, Seongsik Kang, Hae Sun Suh, Young-Hoon Kim

**Affiliations:** 1 Department of Neurology, Samsung Medical Center, Sungkyunkwan University School of Medicine, Seoul, South Korea; 2 Department of Cardiology, Samsung Medical Center, Sungkyunkwan University School of Medicine, Seoul, South Korea; 3 Division of Cardiology, Department of Internal Medicine, Dankook University, Chung Nam, South Korea; 4 Division of Cardiology, Department of Internal Medicine, Catholic University of Korea, Seoul, South Korea; 5 Division of Cardiology, Department of Internal Medicine, Dongsan Hospital, Keimyung University School of Medicine, Daegu, South Korea; 6 Pharmaceutical Economics, Outcomes Research & Policy, College of Pharmacy, Pusan National University, Busan, South Korea; 7 Pfizer Korea Ltd., Seoul, South Korea; 8 Division of Cardiology, Department of Internal Medicine, Korea University, Seoul, South Korea; Ohio State University, UNITED STATES

## Abstract

**Background:**

Although randomized trials provide a high level of evidence regarding the efficacy of non-vitamin K oral anticoagulants (NOACs), the results of such trials may differ from those observed in day-to-day clinical practice.

**Aims:**

To compare the risk of stroke/systemic embolism (S/SE) and major bleeding (MB) between NOAC and warfarin in clinical practice.

**Methods:**

Patients with non-valvular atrial fibrillation (NVAF) who started warfarin/NOACs between January 2015 and November 2016 were retrospectively identified from Korea’s nationwide health insurance claims database. Using inpatient diagnosis and imaging records, the Cox models with inverse probability of treatment weighting using propensity scores were used to estimate hazard ratios (HRs) for NOACs relative to warfarin.

**Results:**

Of the 48,389 patients, 10,548, 11,414, 17,779 and 8,648 were administered apixaban, dabigatran, rivaroxaban and warfarin, respectively. Many patients had suffered prior strokes (36.7%, 37.7%, 31.4%, and 32.2% in apixaban, dabigatran, rivaroxaban, and warfarin group, respectively), exhibited high CHA_2_DS_2_-VASc (4.8, 4.6, 4.6, and 4.1 in apixaban, dabigatran, rivaroxaban, and warfarin group, respectively) and HAS-BLED (3.7, 3.6, 3.6, and 3.3 in apixaban, dabigatran, rivaroxaban, and warfarin group, respectively) scores, had received antiplatelet therapy (75.4%, 75.7%, 76.8%, and 70.1% in apixaban, dabigatran, rivaroxaban, and warfarin group, respectively), or were administered reduced doses of NOACs (49.8%, 52.9%, and 42.8% in apixaban, dabigatran, and rivaroxaban group, respectively). Apixaban, dabigatran and rivaroxaban showed a significantly lower S/SE risk [HR, 95% confidence intervals (CI): 0.62, 0.54–0.71; 0.60, 0.53–0.69; and 0.71, 0.56–0.88, respectively] than warfarin. Apixaban and dabigatran (HR, 95% CI: 0.58, 0.51–0.66 and 0.75, 0.60–0.95, respectively), but not rivaroxaban (HR, 95% CI: 0.84, 0.69–1.04), showed a significantly lower MB risk than warfarin.

**Conclusions:**

Among Asian patients who were associated with higher bleeding risk, low adherence, and receiving reduced NOAC dose than that provided in randomised controlled trials, all NOACs were associated with a significantly lower S/SE risk and apixaban and dabigatran with a significantly lower MB risk than warfarin.

## Introduction

Large randomized controlled trials (RCTs) of patients with non-valvular atrial fibrillation (NVAF) have established that non-vitamin K oral anticoagulants (NOACs) are as effective as warfarin for preventing stroke/systemic embolism (S/SE) and safer than warfarin with regard to major bleeding (MB) and intracranial hemorrhage (ICH) [1–4], making NOACs the recommended first-line drugs for treating stroke prophylaxis in patients with NVAF, and their use has grown dramatically worldwide [5–7].

Although RCTs have provided a high level of evidence regarding the efficacy of NOACs, the results of such trials may differ from those observed in day-to-day clinical practice. This can arise when the patients enrolled in RCTs do not represent those encountered in clinical practice. For instance, the trials included only a relatively small number of Asian patients, which may be of relevance because warfarin-associated ICH is more prevalent in Asian populations and anticoagulation control quality can be worse than that for Caucasian populations [8, 9]. In reality, an RCT in Japan demonstrated that rivaroxaban at a reduced dose of 10–15 mg/day was effective and safe, which is now the common dose administered in countries such as Japan and Taiwan [10, 11].

There is limited evidence based on data from clinical practice on the effectiveness and safety of NOACs compared to warfarin; moreover, assessment of the optimal anticoagulant therapy for patients with atrial fibrillation (AF) based on actual clinical practice data that is country-specific is definitely needed. We hypothesized that NOACs are more effective and safer than warfarin for patients with AF in clinical settings where the quality of care may not be as good as that in RCTs. Thus, this study compared the thromboembolic events and MB/ICH in Korean patients with NVAF treated with NOACs (dabigatran/rivaroxaban/apixaban) and those treated with warfarin, based on Korea’s nationwide insurance claims database.

## Materials and methods

### Data source

The study data were obtained from the Korean Health Insurance Review and Assessment Service (HIRA) database for the period of January 2007 to November 2016. South Korea has a universal health insurance system; thus, this database covers the entire population [12]. The data included the baseline demographics and medical and pharmacy claims of the patients, which were anonymously provided [12]. Diagnoses in the database were coded according to the Korean Standard Classification of Diseases, which is based on the *International Classification of Diseases 10*^*th*^ *Revision* (ICD-10) codes [12]. The Pusan National University Institutional Review Board determined that this study was exempted from ethical review (PNU IRB/2016_137_HR).

### Study population

The study included oral anticoagulant (OAC)-naïve patients who received at least one prescription for apixaban/dabigatran/rivaroxaban/warfarin during the intake period (1 July 2015 to 30 November 2016). The date of the first prescription of OAC was designated as the index date. The inclusion criteria were as follows: age ≥18 years on the index date and at least two outpatient visits or hospitalization at least once with a diagnosis of AF (ICD-10 code I48) before or on the index date. These criteria had performed well in a previous validation study with a positive predictive value (94.1%) [13]. The exclusion criteria were as follows: hip/knee replacement surgery within 6 weeks prior to the index date, valvular AF/a prosthetic heart valve/venous thromboembolism/thyrotoxicosis/hypertrophic cardiomyopathy/elective defibrillation/radiofrequency ablation/left atrial appendage occlusion/end-stage chronic kidney disease/kidney transplant/dialysis/pericarditis during the 12-month baseline period, or prescriptions for more than one OAC on the index date.

Patient data were followed from the index date to the date of the first occurrence of one of the following: switching from the index treatment to another OAC, discontinuation, death or 30 November 2016. Discontinuation was defined as no prescription for any OAC treatment within 30 days after the last day of supply of the final fulfilled prescription. Switching was defined as a prescription for non-index OAC treatment within 30 days after the last day of supply of the last filled prescription or during the index OAC treatment use after the index date.

### Study endpoints

The effectiveness outcome was S/SE, including ischemic and haemorrhagic stroke and SE. The safety outcome was MB, including ICH, gastrointestinal (GI) and other bleeding.

Stroke and ICH events were identified from the diagnosis codes using hospitalization and brain computed tomography/magnetic resonance imaging (CT/MRI) records [14, 15]. SE events were identified from diagnosis codes with hospitalization and any CT/MRI records. GI and other bleeding events were identified from the related diagnosis codes using hospitalization records. In defining each outcome, we used the primary and all secondary diagnosis codes. Further details are provided in S1 Table in S1 File.

### Statistical analysis

To balance baseline demographics and clinical characteristics, we applied the inverse probability of treatment weighting (IPTW) method using the propensity score. Because the weights may be unstable for patients who received treatment contrary to prediction, we used stabilized weights [16]. Propensity scores were estimated using logistic regression that included age, sex, insurance type, baseline medication use, baseline comorbidities (heart failure, hypertension, diabetes, ischemic stroke, vascular disease, abnormal renal function, abnormal liver function, bleeding, alcoholism, and chronic pulmonary disease), Charlson comorbidity index (CCI), CHA_2_DS_2_-VASc score, HAS-BLED score and individual risk factors of the CHA_2_DS_2_-VASc and HAS-BLED scores. We evaluated the balance between the treatment groups using standardized differences in the weighted sample [17]. A standardized difference of <10% was considered acceptable [17].

To address confounding in observational studies, Propensity Score (PS) based methods such as matching, stratification, and IPTW are widely used. For this study, we used IPTW for the following reasons. First, IPTW performed better to balance baseline characteristics between treatment and control group compared to matching or stratification when the number of patients in comparison groups largely differ [18]. In this study, the number of patients with warfarin were less than that of NOAC. IPTW also performed better to remove a greater degree of systematic differences between two groups compared to other PS methods [19, 20]. Second, we used the entire population in IPTW thus we did not lose any data, which was the advantage of using IPTW [18]. Usually, some patients could have been excluded under PS matching method because they were not matched. Third, we did not have a concern with extreme weight problem, which is a statistical term referring to cases when there are patients who received treatment contrary to prediction. IPTW results are generally thought to be unstable under extreme weight problem. Since the results did not change when we trimmed 1% and 0.5% of both sides of distribution of weight, we concluded that an extreme weight problem would not be an issue in this study.

Cox proportional hazards models were used to compare the outcomes between the treatment groups, calculating the hazard ratios (HRs) with 95% confidence intervals (CIs). The proportional hazard assumption was assessed using log–log plots, Schoenfeld’s residuals and time-dependent covariates [21]; however, if this assumption was not met, an extended Cox model that included the product term of the variable with a log of time was used to estimate the HR at 1 year [21]. Each patient’s medication adherence was assessed by calculating the medication possession ratio from the total days of index treatment prescribed medication during 1 year of follow-up [22].

Subgroup analyses were performed based on patient baseline characteristics, including the CHA_2_DS_2_-VASc score, HAS-BLED score, age and sex. Diagnosis and medication codes used for the analysis are provided in S2–S6 Tables in S1 File. We used SAS 9.4 (SAS Institute Inc., Cary, NC, USA) for all analyses.

### Sensitivity analysis

For the first sensitivity analysis, we assessed the crude event rates by restricting the ischemic stroke events to those that met all of the following criteria: inpatient claims with ischemic stroke (ICD-10 code I63/I693/G459) as the primary diagnosis, claims at the Department of Neurology and Neurosurgery and claims with brain CT/MRI records. For the second sensitivity analysis, we assessed the crude event rates by restricting the ischemic stroke and haemorrhagic stroke events to those that met all of the following criteria: inpatient claims with ischemic stroke (ICD-10 code I63/G459) or hemorrhagic stroke (ICD-10 code I61/I62) as the primary diagnosis, claims at the Department of Neurology and Neurosurgery and claims with brain CT/MRI records.

## Results

### Baseline characteristics

**Fig 1:**
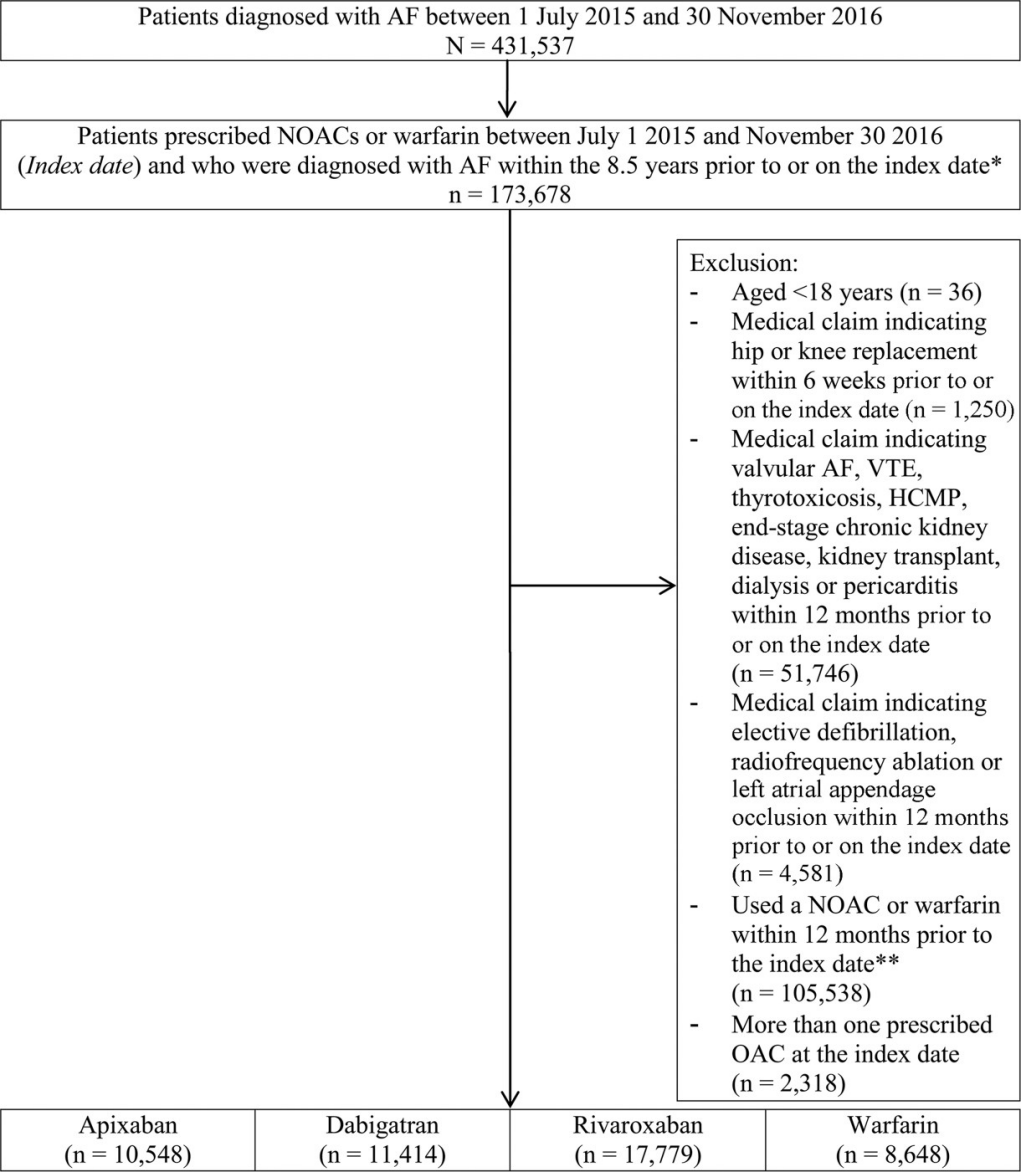
Cohort creation flowchart. AF, atrial fibrillation; HCMP, hypertrophic cardiomyopathy; ICD-10, *International Classification of Diseases 10*^*th*^ *Revision*; NOAC, non-vitamin K antagonist oral anticoagulant; OAC, oral anticoagulant; VTE, venous thromboembolism. * Patients were required to have made at least one inpatient claim/two outpatient claims with a diagnosis of ICD-10 code I48 (atrial fibrillation and flutter) within 8.5 years prior to or on the index date. ** No OACs (apixaban/dabigatran/rivaroxaban/warfarin) should have been prescribed 1 year prior to the index date.

Of the 48,389 OAC-naïve patients identified from the HIRA database, 10,548, 11,414, 17,779 and 8,648 were prescribed apixaban, dabigatran, rivaroxaban (regardless of doses) and warfarin, respectively (Fig 1). Before the weighting application, patients who started warfarin were younger with lower CHA_2_DS_2_-VASc, HAS-BLED, and CCI scores than those who started apixaban/dabigatran/rivaroxaban. After applying weighting using the IPTW method, differences in baseline characteristics were balanced (all *P* > 0.05; absolute standardized difference < 0.1; Table 1).

**Table 1:** Baseline characteristics of patients who were prescribed warfarin and NOACs.

	Inverse probability of treatment weighting											
	Before						After^1^^,^^2^^)^					
	Apixaban (n = 10,548)	Warfarin (n = 8,648)	Dabigatran (n = 11,414)	Warfarin (n = 8,648)	Rivaroxaban (n = 17,779)	Warfarin (n = 8,648)	Apixaban	Warfarin	Dabigatran	Warfarin	Rivaroxaban	Warfarin
Age (years), mean	73.89	68.73	72.23	68.73	73.27	68.73	71.64	71.64	70.68	70.78	71.83	71.85
Female, %	48.81	37.44	43.16	37.44	45.95	37.44	43.93	44.11	41.01	40.84	43.15	43.47
Insurance, %												
National health insurance	93.32	92.45	92.10	92.45	92.33	92.45	92.63	92.85	92.22	92.28	92.38	92.45
Medical aid	6.68	7.55	7.90	7.55	7.67	7.55	7.37	7.15	7.78	7.72	7.62	7.55
CHADS_2_, mean	3.15	2.75	3.05	2.75	3.00	2.75	3.02	3.01	2.95	2.96	2.94	2.98
CHA_2_DS_2_-VASc, mean	4.78	4.06	4.58	4.06	4.60	4.06	4.52	4.51	4.40	4.41	4.45	4.49
HAS-BLED, mean	3.65	3.34	3.58	3.34	3.58	3.34	3.54	3.53	3.50	3.50	3.52	3.53
CCI, mean	4.33	4.04	4.05	4.04	4.11	4.04	4.25	4.25	4.10	4.09	4.11	4.16
Medical history, %												
Heart failure	43.82	41.00	40.96	41.00	43.90	41.00	43.19	42.94	41.18	41.11	43.07	43.36
Hypertension	88.49	80.80	89.18	80.80	89.81	80.80	85.72	85.34	85.74	85.81	87.10	87.12
Diabetes	56.74	51.57	54.89	51.57	54.61	51.57	55.27	55.21	53.92	54.19	53.99	54.82
Ischemic stroke	36.73	32.15	37.69	32.15	31.37	32.15	36.09	35.91	36.59	36.73	32.38	33.38
Vascular disease	29.69	29.24	29.16	29.24	30.06	29.24	29.47	29.70	29.46	29.29	29.95	30.28
Renal disease (CKD3/4)	2.17	2.45	0.91	2.45	1.45	2.45	2.31	2.30	1.83	1.58	1.83	1.83
Bleeding	12.80	13.54	8.73	13.54	10.25	13.54	13.15	13.23	11.16	10.90	11.52	11.59
Cancer	11.24	7.96	9.46	7.96	10.42	7.96	11.04	8.15	9.80	7.55	10.48	7.80
Medication history, %												
NSAIDs	80.93	76.38	81.21	76.38	82.20	76.38	78.92	78.80	78.84	79.01	80.17	80.18
Antiplatelets	75.44	70.06	75.67	70.06	76.80	70.06	73.21	73.34	73.64	73.72	74.58	74.53
Antiarrhythmics	52.25	48.60	47.30	48.60	42.62	48.60	50.54	50.58	48.09	47.82	44.48	44.52
Statins	59.74	51.41	61.47	51.41	55.75	51.41	56.94	56.77	57.50	57.79	54.80	55.45
PPI	45.05	42.77	42.89	42.77	42.50	42.77	44.32	44.40	42.79	42.88	42.71	42.86
H2RA	68.89	66.27	68.71	66.27	67.45	66.27	68.04	67.83	68.04	67.83	67.17	67.41
Digoxin	23.79	27.56	25.03	27.56	26.01	27.56	25.80	25.68	26.13	26.24	26.56	26.78

CCI, Charlson Comorbidity Index; CHADS_2_, score based on congestive heart failure, hypertension, age ≥75 years, diabetes mellitus, prior stroke or transient ischemic attack; CHA_2_DS_2_-VASc, score based on congestive heart failure, hypertension, age ≥75 years, diabetes mellitus, stroke, vascular disease, age 65–74 years and sex; CKD, chronic kidney disease; HAS-BLED, score based on hypertension, abnormal renal and liver function, stroke, bleeding, labile international normalized ratio, elderly, drugs or alcohol; H2RA, H_2_-receptor antagonists; PPI, proton pump inhibitors.

1) *P* values were not significant for all the comparisons (apixaban vs. warfarin; dabigatran vs. warfarin and rivaroxaban vs. warfarin) for variables used to derive propensity score.

2) Absolute standardized differences were <10% for all the comparisons (apixaban vs. warfarin; dabigatran vs. warfarin and rivaroxaban vs. warfarin) for variables used to derive propensity score.

#### Effectiveness outcomes

**Fig 2:**
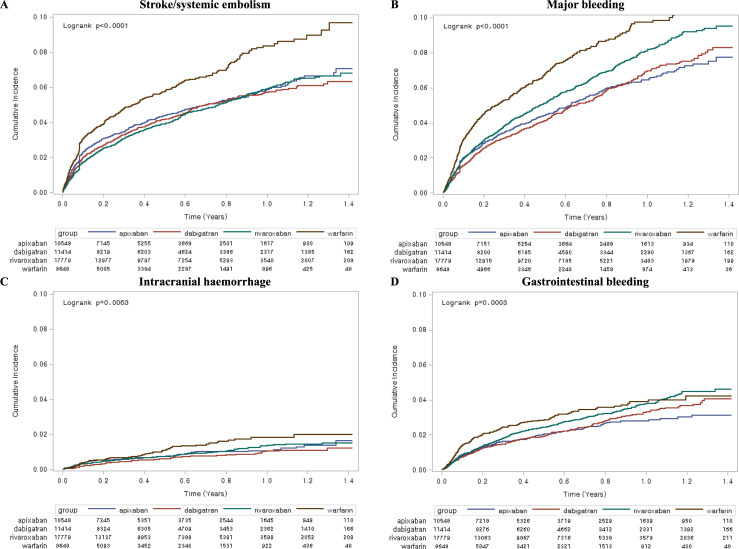
Crude cumulative incidence curves. (A) stroke/systemic embolism, (B) major bleeding, (C) intracranial hemorrhage and (D) gastrointestinal bleeding.

**Fig 3:**
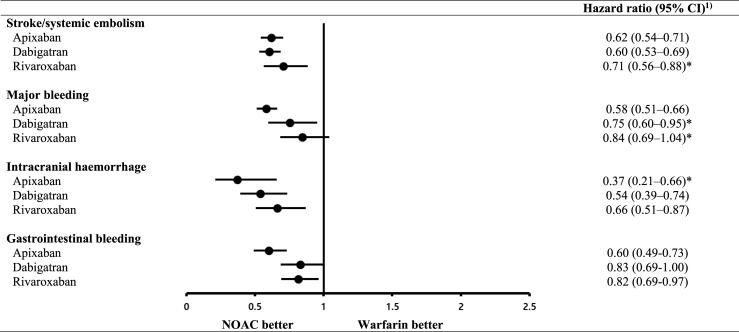
Hazard ratios for stroke/systemic embolism, major bleeding, intracranial haemorrhage and gastrointestinal bleeding for non-vitamin K antagonist oral anticoagulants relative to warfarin. NOACs, non-vitamin K antagonist oral anticoagulants. 1) Obtained using inverse probability of treatment weighting. The patient group treated with warfarin served as the reference. * Reported hazard ratio at 1 year since the proportional hazard assumption was violated.

The crude cumulative incidence curve for S/SE for the patients prescribed warfarin was higher than the corresponding curves for those prescribed NOACs (Fig 2). The weighted event rate for S/SE was 7.2–7.7/100 person-years for the three NOACs and 12.9–13.5/100 person-years for warfarin (Table 2). Apixaban, dabigatran and rivaroxaban were associated with a significantly lower S/SE risk than warfarin, with the following HRs (95% CIs): apixaban, 0.62 (0.54–0.71); dabigatran, 0.60 (0.53–0.69); and rivaroxaban, 0.71 (0.56–0.88) (Fig 3).

**Table 2:** Number of events and crude and adjusted event rates of non-vitamin K antagonist oral anticoagulants or warfarin users.

	Inverse probability of treatment weighting													
	Before								After					
	Apixaban (n = 10,548)		Dabigatran (n = 11,414)		Rivaroxaban (n = 17,779)		Warfarin (n = 8,648)		Apixaban	Warfarin	Dabigatran	Warfarin	Rivaroxaban	Warfarin
	Events	Crude IR^1)^	Events	Crude IR^1)^	Events	Crude IR^1)^	Events	Crude IR^1)^	Adjusted IR^1)^^2)^					
S/SE	419	8.00	451	7.14	700	7.07	413	11.86	7.66	13.52	7.20	13.53	7.18	12.89
MB	432	8.26	491	7.81	927	9.44	466	13.53	7.97	14.73	8.57	13.77	9.55	14.23
ICH	76	1.43	68	1.06	140	1.40	73	2.06	1.37	2.38	1.17	2.31	1.47	2.37
GIb	188	3.55	234	3.68	432	4.33	196	5.57	3.44	6.2	4.17	5.56	4.32	5.78

GIb, GI bleeding; ICH, intracranial haemorrhage; IR, incidence rate; MB, major bleeding; S/SE, stroke/systemic embolism.

1) Incidence rates were calculated as the number of patients experiencing the event divided by 100 person-years.

2) Obtained using inverse probability of treatment weighting.

#### Safety outcomes

The crude cumulative incidence curves for MB and ICH for the patients prescribed warfarin were higher than the corresponding curves for those who were prescribed NOACs (Fig 2). The weighted event rate for MB was 8.0–9.6/100 person-years for the three NOACs and 13.8–14.7/100 person-years for warfarin (Table 2). The crude event rates of GI bleeding were 3.55, 3.68, 4.33, and 5.57/100 person-years in apixaban, dabigatran, rivaroxaban, and warfarin group, respectively. The weighted event rates of GI bleeding for all NOACs were lower versus warfarin: 3.44 and 6.20/100 person-years in apixaban and warfarin group, respectively; 4.17 and 5.56/100 person-years in dabigatran and warfarin group, respectively; 4.32 and 5.78/100 person-years in rivaroxaban and warfarin group, respectively (Table 2).

Compared to warfarin, apixaban and dabigatran were associated with significantly lower MB and ICH risks, whereas rivaroxaban was associated with a significantly lower ICH risk, but not MB risk. The HRs (95% CIs) were as follows: for MB, apixaban, 0.58 (0.51–0.66); dabigatran, 0.75 (0.60–0.95); and rivaroxaban, 0.84 (0.69–1.04) and for ICH, apixaban, 0.37 (0.21–0.66); dabigatran, 0.54 (0.39–0.74); and rivaroxaban, 0.66 (0.51–0.87) (Fig 3). Compared to warfarin, the HRs (95% CIs) for GI bleeding were apixaban, 0.60 (0.49–0.73); dabigatran, 0.83 (0.69–1.00); and rivaroxaban, 0.82 (0.69–0.97) (Fig 3).

#### Subgroup analyses

**Fig 4:**
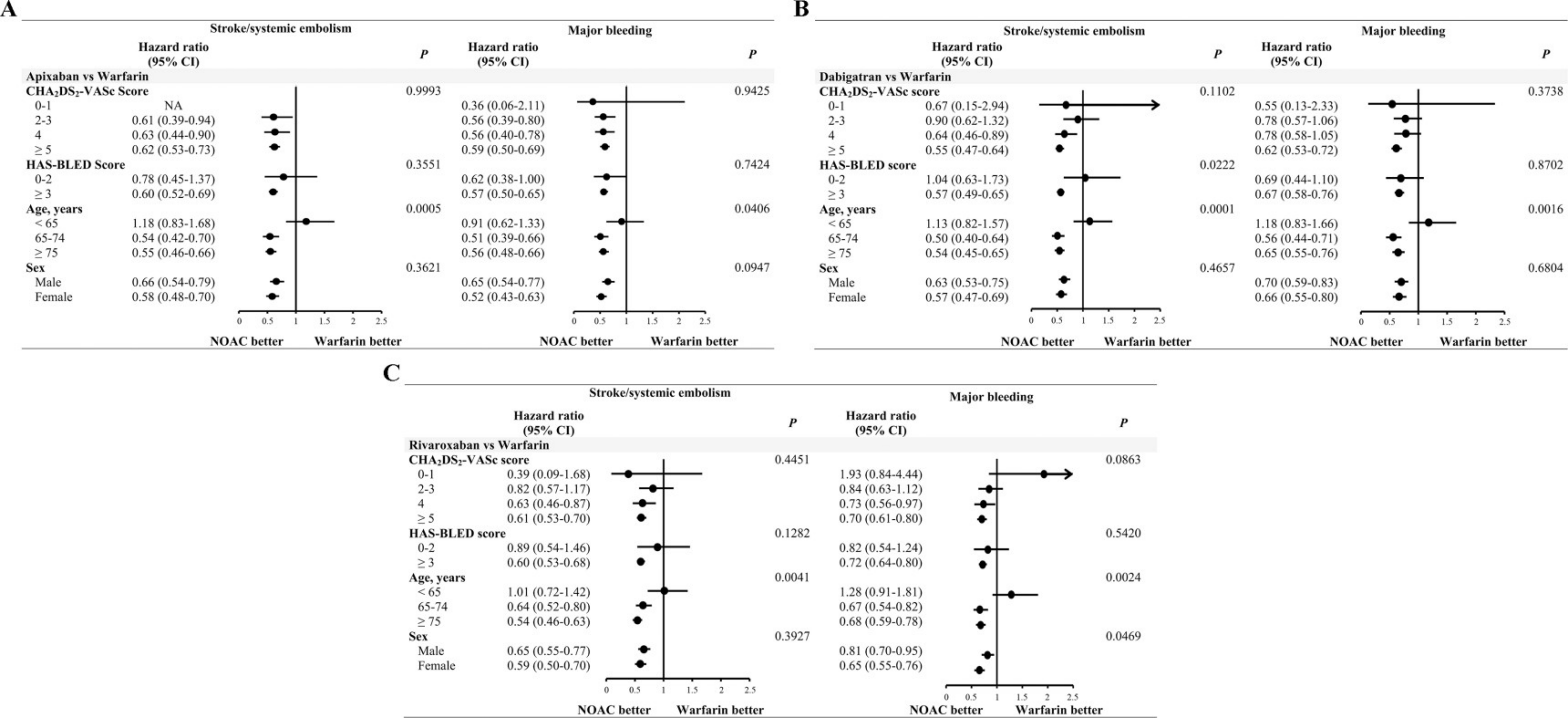
Subgroup analyses for the comparisons of stroke/systemic embolism and major bleeding between the three non-vitamin K antagonist oral anticoagulants and warfarin. (A) apixaban vs warfarin, (B) dabigatran vs warfarin, (C) rivaroxaban vs warfarin. CHA_2_DS_2_-VASc, score based on congestive heart failure, hypertension, age ≥75 years, diabetes mellitus, stroke, vascular disease, age 65–74 years and sex; CI, confidence interval; CKD, chronic kidney disease; HAS-BLED, score based on hypertension/abnormal renal and liver function/stroke/bleeding/labile international normalized ratio/elderly/drugs/alcohol; NOACs, non-vitamin K antagonist oral anticoagulants. The *P* value is for the interaction.

In the subgroup analyses for the comparisons of the three NOACs with warfarin, no interactions with the treatment effect were observed for the subgroups of CHA_2_DS_2_-VASc score, HAS-BLED score and sex, except for that of age (Fig 4).

#### Sensitivity analyses

The crude event rates in the two sensitivity analyses, in which the stroke events were restricted to those that met stricter definitions, were lower overall than those of the main analysis (Table 3).

**Table 3:** Summary of the crude event rates for stroke in the main analysis and the two sensitivity analyses.

	Main analysis				Sensitivity analysis 1^1^^)^				Sensitivity analysis 2^2^^)^			
	Apixaban (n = 10,548)	Dabigatran (n = 11,414)	Rivaroxaban (n = 17,779)	Warfarin (n = 8,648)	Apixaban (n = 10,548)	Dabigatran (n = 11,414)	Rivaroxaban (n = 17,779)	Warfarin (n = 8,648)	Apixaban (n = 10,548)	Dabigatran (n = 11,414)	Rivaroxaban (n = 17,779)	Warfarin (n = 8,648)
	Crude incidence rates				Crude incidence rates				Crude incidence rates			
IS	7.25	6.54	6.20	10.33	2.91	3.02	3.11	5.58	2.89	3.02	3.09	5.52
HS	1.13	0.69	1.12	1.52	NA	NA	NA	NA	0.41	0.09	0.56	0.65

HS, haemorrhagic stroke; ICD-10, *International Classification of Diseases 10*^*th*^ *Revision*; IS, ischemic stroke; NA, not assessed.

Incidence rates were calculated as the number of patients with the event divided by 100 person-years.

1) In sensitivity analysis 1, ischemic stroke events were restricted to those meeting all of the following criteria: an inpatient claim for ischemic stroke (ICD-10 code I63, I693 or G459) as the primary diagnosis, a claim at the Department of Neurology and Neurosurgery and brain CT/MRI records.

2) In sensitivity analysis 2, the ischemic stroke and haemorrhagic stroke events were restricted to those meeting all of the following criteria: an inpatient claim for ischemic stroke (ICD-10 code I63 or G459) or haemorrhagic stroke (ICD-10 code I61 or I62) as the primary diagnosis, a claim at the Department of Neurology and Neurosurgery and brain CT/MRI records.

## Discussion

We found that patients in real-world clinical practice showed different characteristics and received a different quality of care than those enrolled in RCTs. In addition, all three NOACs were significantly associated with a reduced S/SE risk than with warfarin, and apixaban and dabigatran were associated with a significantly reduced MB risk than with warfarin in these patients.

Evidence from clinical practice and RCTs is considered to be mutually complementary. Therefore, results from actual clinical practice may help in translating RCT results into protocols and guidelines for health professionals to apply in their own clinical practice. Many patients with NVAF are prescribed multiple medications, and antiplatelet agents are widely used in clinical practice. The concomitant use of antiplatelet agents with oral anticoagulants can increase ICH and MB risks. In one RCT, 34% of the warfarin and NOAC groups used aspirin at baseline [23]. In this study, approximately three-quarters of the patients received both antiplatelet therapy and oral anticoagulants.

Observational studies in Asian countries have reported that a large proportion of patients with AF is being sub-optimally treated with NOACs [11]. In this study, approximately half of the patients were initiated a reduced dose of NOACs. The inappropriate use of reduced NOAC doses may reduce bleeding event risk; however [24], there are controversial results from Asian countries about the stroke event risk with the use of reduced NOAC doses than standard doses [25–27]. A few retrospective, nationwide cohort studies from the USA and Europe have shown worse effectiveness outcomes with reduced NOAC doses than with warfarin [28, 29]. Moreover, the results of this study should be interpreted with caution because whether dose selection matches indicated dose reduction criteria was unclear from the data source.

The effectiveness and safety may be influenced by the patient’s thromboembolic risk and prior stroke. In this study, the mean CHADS_2_ scores were similar to those of four pivotal RCTs of NOACs [1–4]. Approximately one-third of the patients in this study had suffered a stroke, whereas a prior stroke/transient ischemic attack was reported for about one-fifth of the patients in the RE-LY, ARISTOTLE and ENGAGE AF-TIMI 48 trials and in half of the patients in the ROCKET-AF trial [1–4].

Given the absence of routine anticoagulation monitoring with NOAC treatment, adherence with the treatment protocol is paramount for the efficacy and safety of all oral anticoagulants [30, 31]. In this study, the 1-year medication possession ratio, an indicator of medication adherence, was 0.42 and 0.70 for warfarin and NOACs, respectively. It was impossible to make a direct comparison with the RCTs of NOACs, which reported only discontinuation rates and not adherence per se, and claims-based/patient-reported methods to measure oral anticoagulation adherence may lead to different results [31, 32]. Anticoagulation control with warfarin may be lower for patients in real clinical practice than for those included in RCTs. In the aforementioned RCTs, the median time within the therapeutic range was 58%–68%, and based on a multicentre study including Korean patients, it was at a much lower range (49.1%) [33].

Recently, there have been reports of several nationwide population-based studies conducted in Korea, Taiwan, USA and France and of prospective post-marketing safety surveillance studies; these are summarized in S7 Table in S1 File. Several studies reported that NOACs were associated with better effectiveness and safety than warfarin [25, 27, 34], whereas other studies showed inconclusive results, such as dabigatran having similar effectiveness, reduced ICH and increased MB vs. warfarin [35], or no differences in effectiveness and safety between dabigatran/rivaroxaban and warfarin [36]. These may be cautiously interpreted with the differences in patient characteristics (ethnicity/comorbidities/quality of control), the coding system used for comorbidities and outcomes, or the dose/type of NOAC used in each study. This study showed that all three NOACs were associated with lower S/SE and ICH risks than warfarin in Asian patients with AF who were associated with higher bleeding risk, low adherence, and reduced NOAC dose intake.

The present study had several strengths. First, the data source was Korea’s HIRA claims database, which includes data of the entire Korean population and provides opportunities to study the interventional effectiveness in the general population in routine clinical practice, including populations who may be excluded from/underrepresented in RCTs and other claims databases [12]. Second, this study has two sensitivity analyses for stroke. We used inpatient diagnosis codes in any position to identify outcomes. This may complicate the comparison of the results with those of previous studies that used only primary diagnosis codes for their outcomes. Our two sensitivity analyses showed incidence rates that were generally similar to those reported in two Taiwanese studies using only primary diagnosis codes [11, 27]. This indicates that the diagnosis codes are important in the outcome definition and that some outcomes may be missed if defined only by the primary diagnosis code.

This study had several limitations inherent to retrospective analyses based on claims data. There may have been other unmeasured confounding or coding errors of comorbidities and outcomes. Moreover, we assumed that the patients took all the medications as prescribed; however, it was impossible to accurately measure whether the patients took their medication or the precise timing of treatment discontinuation. The HIRA database does not contain laboratory data (e.g. international normalized ratio data for patients treated with warfarin) or information about the use of over-the-counter medication. Lastly, the follow-up period (median follow-up time were 149, 171, 175, and 105 days in apixaban, dabigatran, rivaroxaban, and warfarin group, respectively) was relatively short because insurance reimbursement expansion of NOAC as first-line therapy took effect from 1 July 2015. Thus, further studies may be required to explore the long-term effectiveness and safety of NOACs.

## Supporting information

S1 File (DOCX)
