# Supplementary material for: The risk of stroke/systemic embolism and major bleeding in Asian patients with non-valvular atrial fibrillation treated with non-vitamin K oral anticoagulants compared to warfarin: Results from a real-world data analysis
Source: PLoS One. 2020 Nov 30;15(11):e0242922. doi: 10.1371/journal.pone.0242922 (PMC7703907; doi:10.1371/journal.pone.0242922)
Supplement: S1 File — (DOCX) [file pone.0242922.s001.docx]

The risk of stroke/systemic embolism and major bleeding in Asian patients with non-valvular atrial fibrillation treated with non-vitamin K oral anticoagulants compared to warfarin: results from a real-world data analysis

Oh Young Bang^a^, Young Keun On^b^, Myung-Yong Lee^c^, Sung-Won Jang^d^, Seongwook Han^e^, Sola Han^f^, Mi-Mi Won^g^, Yoo-Jung Park^g^, Ji-Min Lee^g^, Hee-Youn Choi^g^, Seongsik Kang^g^, Hae Sun Suh^f,*^ and Young-Hoon Kim^h,*^

^a^Department of Neurology, Samsung Medical Center, Sungkyunkwan University School of Medicine, Seoul, South Korea

^b^Department of Cardiology, Samsung Medical Center, Sungkyunkwan University School of Medicine, Seoul, South Korea

^c^Division of Cardiology, Department of Internal Medicine, Dankook University, Chung Nam, South Korea

^d^Division of Cardiology, Department of Internal Medicine, Catholic University of Korea, Seoul, South Korea

^e^Division of Cardiology, Department of Internal Medicine, Dongsan Hospital, Keimyung University School of Medicine, Daegu, South Korea

^f^Pharmaceutical Economics, Outcomes Research & Policy, College of Pharmacy, Pusan National University, Busan, South Korea

^g^Pfizer Korea Ltd., Seoul, South Korea

^h^Division of Cardiology, Department of Internal Medicine, Korea University, Seoul, South Korea

* H.S. Suh and Y. Kim are corresponding authors.

**Supplementary Data**

S1 Table. *International Classification of Disease 10^th^ Revision* (ICD-10) codes for the study outcomes

| **Outcomes** | | **ICD-10 codes** |
| --- | --- | --- |
| Stroke or systemic embolism | Ischaemic stroke* | G459, I63 and I693 |
|  | Haemorrhagic stroke* | I60, I61, I62, I690, I691 and I692 |
|  | Systemic embolism** | I74 |
| Major bleeding*** | Intracranial haemorrhage* | I60, I61, I62, I690, I691, I692, S064, S065, S066 and S068 |
|  | Gastrointestinal bleeding | I850, I983, K2211, K226, K228, K250, K252, K254, K256, K260, K262, K264, K266, K270, K272, K274, K276, K280, K282, K284, K286, K290, K3181, K5521, K625, K920, K921 and K922 |
|  | Bleeding from other sites | D62, H448, H3572, H356, H313, H210, H113, H052, H470, H431, I312, N020–N029, N421, N831, N857, N920, N923, N930, N938, N939, M250, R233, R040, R041, R042, R048, R049, T792, T810, N950, R310, R311, R318, R58, T455, Y442 and D683 |
| * Brain CT/MRI procedure codes and hospitalisation were also required to define ischemic stroke, haemorrhagic stroke or intracranial haemorrhage.  ** Any CT/MRI procedure codes and hospitalization were also required to define systemic embolism.  *** Hospitalisation was also required to define major bleeding (i.e. intracranial haemorrhage, gastrointestinal bleeding and bleeding from other sites). | | |

**S2 Table.** Scoring and *International Classification of Disease 10^th^ Revision* (ICD-10) codes for the factors included in the CHA_2_DS_2_-VASc score

| **Condition** | **ICD-10 codes** | **Point** |
| --- | --- | --- |
| Congestive heart failure | I50 | 1 |
| Hypertension | I10–I15 | 1 |
| Age | ≥75 years | 2 |
| Diabetes | E10–E14 | 1 |
| Stroke | I63, I693 and G459 | 2 |
| Vascular disease | I21, I252 and I70–I73 | 1 |
| Age | 65–74 years | 1 |
| Sex | Female | 1 |
|  | | |

**S3 Table.** Scoring and *International Classification of Disease 10^th^ Revision* (ICD-10) codes for the factors included in the HAS-BLED score

| **Condition** | **ICD-10 codes** | **Point** |
| --- | --- | --- |
| Hypertension | I10–I15 | 1 |
| Abnormal renal disease | N183 and N184 | 1 |
| Abnormal liver function | B15–B19, C22, D684, I982, I983, K70–K77 and Z944 | 1 |
| Stroke | I63, I693 and G459 | 1 |
| Bleeding history or predisposition* | I60, I61, I62, I690, I691, I692, S064, S065, S066, S068, I850, I983, K2211, K226, K228, K250, K252, K254, K256, K260, K262, K264, K266, K270, K272, K274, K276, K280, K282, K284, K286, K290, K3181, K5521, K625, K920, K921, K922, D62, H448, H3572, H356, H313, H210, H113, H052, H470, H431, I312, N020-N029, N421, N831, N857, N920, N923, N930, N938, N939, M250, R233, R040, R041, R042, R048, R049, T792, T810, N950, R310, R311, R318, R58, T455, Y442 and D683 | 1 |
| Elderly | ≥65 years | 1 |
| Drug therapy | Antiplatelets and NSAIDs | 1 |
| Alcoholism | E244, F10, G312, G621, G721, I426, K292, K70, K860, O354, P043, Q860, T510, X45, X65, Y15, Y90-Y91, Z502, Z714 and Z721 | 1 |
| * A blood transfusion was also required to define a history of bleeding from other sites. | | |

**S4 Table.** Types of baseline medications for medication history

| **Class** | **Drugs** |
| --- | --- |
| NSAIDs | Bromfenac, celecoxib, diclofenac, etodolac, fenoprofen, flurbiprofen, ibuprofen, indomethacin, ketoprofen, ketorolac, naproxen, meclofenamate, mefenamic acid, meloxicam, nabumetone, oxaprozin, piroxicam, sulindac and tolmetin |
| Antiplatelets | Aspirin, clopidogrel, prasugrel, ticlopidine, cilostazol, abciximab, tirofiban, dipyridamole and ticagrelor |
| Proton pump inhibitors | Omeprazole, pantoprazole, lansoprazole, rabeprazole, esomeprazole and dexlansoprazole |
| H_2_-receptor antagonists | Cimetidine, ranitidine, famotidine, nizatidine, roxatidine and lafutidine |
| Antiarrhythmics | Quinidine, procainamide, mexiletine, propafenone, flecainide, amiodarone, bretylium, dronedarone, propranolol, atenolol, esmolol, verapamil, diltiazem and sotalol |
| Digoxin | Digoxin |
| Statins | Atorvastatin, fluvastatin, lovastatin, pitavastatin, pravastatin, rosuvastatin and simvastatin |

NSAID, non-steroidal anti-inflammatory drug

**S5 Table.** *International Classification of Disease 10^th^ Revision* (ICD-10) codes for medical history

| **Condition** | **ICD-10 codes** |
| --- | --- |
| Heart failure | I50 |
| Hypertension | I10-I15 |
| Diabetes | E10-E14 |
| Ischemic stroke | I63, I693 G459 |
| Vascular disease | I21, I252, I70-I73 |
| Renal disease (CKD3/4) | N183, N184 |
| Bleeding | I60, I61, I62, I690, I691, I692, S064, S065, S066, S068, I850, I983, K2211, K226, K228, K250, K252, K254, K256, K260, K262, K264, K266, K270, K272, K274, K276, K280, K282, K284, K286, K290, K3181, K5521, K625, K920, K921, K922, D62, H448, H3572, H356, H313, H210, H113, H052, H470, H431, I312, N020-N029, N421, N831, N857, N920, N923, N930, N938, N939, M250, R233, R040, R041, R042, R048, R049, T792, T810, N950, R310, R311, R318, R58, T455, Y442, D683 |
| Cancer (any malignancy/metastatic solid tumor) | C00–C26, C30–C34, C37–C41, C43, C45–C58, C60–C76, C81–C85, C88, C90–C97, C77–C80 |

**S6 Table.** Scoring and *International Classification of Disease 10^th^ Revision* (ICD-10) codes for the factors included in the Charlson comorbidity index

| **Condition** | **ICD-10 codes** | **Point** |
| --- | --- | --- |
| Cerebrovascular disease | G45.x, G46.x, H34.0 and I60.x–I69.x | 1 |
| Congestive heart failure | I09.9, I11.0, I13.0, I13.2, I25.5, I42.0, I42.5–I42.9, I43.x, I50.x and P29.0 | 1 |
| Chronic pulmonary disease | I27.8, I27.9, J40.x–J47.x, J60.x–J67.x, J68.4, J70.1 and J70.3 | 1 |
| Dementia | F00.x–F03.x, F05.1, G30.x and G31.1 | 1 |
| Diabetes without chronic complication | E10.0, E10.1, E10.6, E10.8, E10.9, E11.0, E11.1, E11.6, E11.8, E11.9, E12.0, E12.1, E12.6, E12.8, E12.9, E13.0, E13.1, E13.6, E13.8, E13.9, E14.0, E14.1, E14.6, E14.8 and E14.9 | 1 |
| Mild liver disease | B18.x, K70.0–K70.3, K70.9, K71.3–K71.5, K71.7, K73.x, K74.x, K76.0, K76.2–K76.4, K76.8, K76.9 and Z94.4 | 1 |
| Myocardial infarction | I21.x, I22.x and I25.2 | 1 |
| Peripheral vascular disease | I70.x, I71.x, I73.1, I73.8, I73.9, I77.1, I79.0, I79.2, K55.1, K55.8, K55.9, Z95.8 and Z95.9 | 1 |
| Peptic ulcer disease | K25.x–K28.x | 1 |
| Rheumatologic disease | M05.x, M06.x, M32.x–M34.x, M31.5, M35.1, M35.3 and M36.0 | 1 |
| Diabetes with chronic complication | E10.2–E10.5, E10.7, E11.2–E11.5, E11.7, E12.2–E12.5, E12.7, E13.2–E13.5, E13.7, E14.2–E14.5 and E14.7 | 2 |
| Hemiplegia or paraplegia | G04.1, G11.4, G80.1, G80.2, G81.x, G82.x, G83.0–G83.4 and G83.9 | 2 |
| Any malignancy including leukaemia and lymphoma | C00.x–C26.x, C30.x–C34.x, C37.x–C41.x, C43.x, C45.x–C58.x, C60.x–C76.x, C81.x–C85.x, C88.x and C90.x–C97.x | 2 |
| Renal disease | I12.0, I13.1, N03.2–N03.7, N05.2–N05.7, N18.x, N19.x, N25.0, Z49.0–Z49.2, Z94.0 and Z99.2 | 2 |
| Moderate or severe liver disease | I85.0, I85.9, I86.4, I98.2, K70.4, K71.1, K72.1, K72.9, K76.5, K76.6 and K76.7 | 3 |
| AIDS/HIV | B20.x–B22.x and B24.x | 6 |
| Metastatic solid tumour | C77.x–C80.x | 6 |
|  | | |

**S7 Table.** Summary of previous observational studies that investigated the safety and effectiveness of NOACs

| **Reference** | **Database** | **Main findings** |
| --- | --- | --- |
| **Claims database** |  |  |
| Cho MS et al. 2019^1^ | Korean National Health Insurance Service database (n = 56,504) | NOACs were associated with better effectiveness and safety outcomes than warfarin.  Low doses of NOACs were prescribed more frequently than standard doses (75% of dabigatran, 59% of rivaroxaban and 63% of apixaban).  Low-dose apixaban for patients aged <75 years without CKD seemed to reduce clinical benefit. |
| Cha MJ et al. 2017^2^ | Korean National Health Insurance Service database (NOAC, n = 11,611; VKA, n = 23,222) | Compared to VKA, all three NOACs demonstrated a similar risk of IS and a lower risk of ICH.  ICH prevention was superior with dabigatran and apixaban than with warfarin and rivaroxaban.  All-cause mortality was significantly lower only with dabigatran and apixaban. |
| Lee SR et al. 2018^3^ | Korean National Health Insurance Service database (Edoxaban, n = 4,061; VKA, n = 12,184) | Compared to VKA, edoxaban might be associated with a reduced risk of IS, major bleeding and all-cause mortality.  These benefits were consistent across various high-risk sub-groups. |
| Lee KT et al. 2018^4^ | National Health Insurance Research Database of Taiwan (N = 279,576) | CHA2DS2-VASc scores were susceptible to both SE and major bleeding; this trend was consistently observed in patients who consumed NOACs or warfarin. |
| Lin YS et al. 2018^5^ | National Health Insurance Research Database of Taiwan (AF, n = 188,811; A-flutter, n = 6,121; and control, n = 24,484) | CHA2DS2-VASc scores were higher for patients with AF than those for patients with A-flutter or the control group.  For patients with A-flutter, the incidence of IS was only higher with CHA2DS2-VASc scores of 5–9. |
| Chan YH et al. 2018^6^ | National Health Insurance Research Database of Taiwan (N = 279,776) | All NOACs at both low and standard doses were associated with lower risks of IS/SE, major bleeding and mortality than that associated with VKA. |
| Chao TF et al. 2018^7^ | National Health Insurance Research Database of Taiwan (N = 181,214 patients newly diagnosed with AF and with CHA2DS2-VASc scores ≥1 for men and ≥2 for women) | A more than two-fold increase in the prescription rates of OACs was observed after NOACs were clinically introduced.  During the study period, CHA2DS2-VASc scores increased; however, the IS rate decreased without an increase in ICH rate.  The risk of IS was significantly lower in 2012–2015 than in 2008. |
| Graham DJ et al. 2015^8^ | Medicare data for dabigatran or VKA for AF (N = 134,414) | Dabigatran was associated with reduced risks of IS, ICH and death but an increased risk of major GI bleeding.  These associations were pronounced in patients treated with 150 mg BID; at 75 mg BID, the associations were indistinguishable from those of VKA, except for a lower risk of ICH. |
| Lip GYH et al. 2018^9^ | Retrospective observational study of pooled Medicare and Medicaid Services data and four US commercial claim databases (ARISTOPHANES study, N = 285,292) | NOACs were associated with lower rates of IS/SE and major bleeding than VKA. |
| Noseworthy PA et al. 2016^10^ | US administrative claims database (R vs. D, n = 31,574; A vs. D, n = 13,084; A vs. R, n = 13,130) | No differences among the three NOACs regarding the risk of stroke or SE.  Apixaban was associated with a lower risk of major bleeding than other NOACs.  Rivaroxaban was associated with an increased risk of major bleeding and ICH than dabigatran. |
| Yao X, 2016^11^ | Insurance database, privately insured and Medicare (A vs W, n = 15,390; R vs W, n = 32,350; D vs W, n = 28,614) | Compared to warfarin, apixaban was associated with lower risks of stroke and major bleeding, dabigatran was associated with a similar risk of stroke but a lower risk of major bleeding and rivaroxaban was associated with similar risks of both stroke and major bleeding. |
| Maura G et al. 2015^12^ | French National Health Insurance information system and French Hospital Discharge database (dabigatran, n = 8,443; rivaroxaban, n = 4,651; VKA, n = 19,713) | There was no significant difference in bleeding or thromboembolism between VKA and NOACs. |
| **Prospective registries** | |  |
| Oldgren J et al. 2014^13^ | RE-LY AF registry, an international prospective registry that enrolls patients presented to an emergency department with AF (N = 15,400) | Global variation was observed in risk factors, concomitant diseases and AF treatment.  Rheumatic heart disease was prevalent in Africa and India but rare in North America.  The mean time in therapeutic range was 62% in Western Europe and 51% in North America but only 32% in Asia and 40% in Africa. |
| De Caterina R et al. 2014^14^ | PREFER in the AF registry, an international (European) prospective registry (N = 7,243) | The combined use of OACs and antiplatelet therapy was common for AF but largely inappropriate, explained by the coexistence of coronary or peripheral arterial disease. |
| Kirchhof P et al. 2018^15^ | Pooled analysis of the XANTUS, XANAP and XANTUS-EL studies (N = 11,121) | Low bleeding and stroke rates in rivaroxaban-treated patients with AF with low-treatment discontinuation rates in different regions of the world.  The prescription of reduced doses of rivaroxaban was 15%–20% in Western countries but >50% in East Asian countries. |
| Nielsen PB, 2017^16^ | Three nationwide registries in Denmark (N = 55,644) | Apixaban 2.5 mg BID was associated with a trend towards higher rates of IS/SE compared to warfarin, whereas rivaroxaban 15 mg once a day and dabigatran 110 mg BID showed a trend towards lower thromboembolic rates. Rates of bleeding were significantly lower for dabigatran, but not for the other NOACs. |
| **The present study** | Korean Health Insurance Review and Assessment Service (N = 48,389) | Compared to warfarin, all three NOACs were associated with lower risk of S/SE, and apixaban and dabigatran were associated with a lower risk of MB. |

A, apixaban; AF, atrial fibrillation; D, dabigatran; E, edoxaban; ICH, intracranial haemorrhage; IS, ischemic stroke; MB, major bleeding; NOACs; non-vitamin K oral anticoagulants; OACs, oral anticoagulants; R, rivaroxaban; RCT, randomized controlled trial; SE, systemic embolism; VKA, vitamin K anticoagulant; W, warfarin

**References**

1. Cho MS, Yun JE, Park JJ, Kim YJ, Lee J, Kim HM, et al. Outcomes after use of standard- and low-dose non-vitamin K oral anticoagulants in Asian patients with atrial fibrillation. Stroke. 2018:STROKEAHA118023093.

2. Cha MJ, Choi EK, Han KD, Lee SR, Lim WH, Oh SI, et al. Effectiveness and safety of non-vitamin K antagonist oral anticoagulants in Asian patients With atrial fibrillation. Stroke 2017;48: 3040–3048.

3. Lee SR, Choi EK, Han KD, Jung JH, Oh SI, Lip GYH. Edoxaban in Asian patients with atrial fibrillation: effectiveness and safety. J Am Coll Cardiol. 2018;72: 838–853.

4. Lee KT, Chang SH, Yeh YH, Tu HT, Chan YH, Kuo CT, et al. The CHA2DS2-VASc score predicts major bleeding in non-valvular atrial fibrillation patients who take oral anticoagulants. J Clin Med. 2018;7: 338.

5. Lin YS, Chen YL, Chen TH, Lin MS, Liu CH, Yang TY, et al. Comparison of clinical outcomes Among patients with atrial fibrillation or atrial flutter stratified by CHA2DS2-VASc score. JAMA. Netw Open 2018;1: e180941.

6. Chan YH, See LC, Tu HT, Yeh YH, Chang SH, Wu LS, et al. Efficacy and safety of apixaban, dabigatran, rivaroxaban, and warfarin in Asians with nonvalvular atrial fibrillation. J Am Heart Assoc. 2018;7.

7. Chao TF, Chiang CE, Lin YJ, Chang SL, Lo LW, Hu YF, et al. Evolving changes of the use of oral anticoagulants and outcomes in patients with newly diagnosed atrial fibrillation in Taiwan. Circulation. 2018;138: 1485–1487.

8. Graham DJ, Reichman ME, Wernecke M, Zhang R, Southworth MR, Levenson M, et al. Cardiovascular, bleeding, and mortality risks in elderly Medicare patients treated with dabigatran or warfarin for nonvalvular atrial fibrillation. Circulation. 2015;131: 157–164.

9. Lip GYH, Keshishian A, Li X, Hamilton M, Masseria C, Gupta K, et al. Effectiveness and safety of oral anticoagulants Among nonvalvular atrial fibrillation patients. Stroke. 2018; 49:2933–2944.

10. Noseworthy PA, Yao X, Abraham NS, Sangaralingham LR, McBane RD, Shah ND. Direct comparison of dabigatran, rivaroxaban, and apixaban for effectiveness and safety in nonvalvular atrial fibrillation. Chest. 2016;150: 1302–1312.

11. Yao X, Abraham NS, Sangaralingham LR, Bellolio MF, McBane RD, Shah ND, et al. Effectiveness and safety of dabigatran, rivaroxaban, and apixaban versus warfarin in nonvalvular atrial fibrillation. J Am Heart Assoc. 2016;5.

12. Maura G, Blotière PO, Bouillon K, Billionnet C, Ricordeau P, Alla F, et al. Comparison of the short-term risk of bleeding and arterial thromboembolic events in nonvalvular atrial fibrillation patients newly treated with dabigatran or rivaroxaban versus vitamin K antagonists: a French nationwide propensity-matched cohort study. Circulation. 2015;132: 1252–1260.

13. Oldgren J, Healey JS, Ezekowitz M, Commerford P, Avezum A, Pais P, et al. Variations in cause and management of atrial fibrillation in a prospective registry of 15,400 emergency department patients in 46 countries: the RE-LY Atrial Fibrillation Registry. Circulation. 2014; 129:1568–1576.

14. De Caterina R, Ammentorp B, Darius H, Le Heuzey JY, Renda G, Schilling RJ, et al. PREFER in AF Registry Investigators. Frequent and possibly inappropriate use of combination therapy with an oral anticoagulant and antiplatelet agents in patients with atrial fibrillation in Europe. *Heart* 2014;100: 1625–1635.

15. Kirchhof P, Radaideh G, Kim YH, Lanas F, Haas S, Amarenco P, et al. Global XANTUS program Investigators. Global prospective safety analysis of Rivaroxaban. *J Am Coll Cardiol* 2018;72: 141–153.

16. Nielsen PB, Skjøth F, Søgaard M, Kjældgaard JN, Lip GY, Larsen TB. Effectiveness and safety of reduced dose non-vitamin K antagonist oral anticoagulants and warfarin in patients with atrial fibrillation: propensity weighted nationwide cohort study. *BMJ* 2017;356: j510.

17. Ntaios G, Papavasileiou V, Makaritsis K, Vemmos K, Michel P, Lip GYH. Real-world setting comparison of nonvitamin-K antagonist oral anticoagulants versus vitamin-K antagonists for stroke prevention in atrial fibrillation: A systematic review and meta-analysis. Stroke. 2017;48: 2494–2503.

18. Proietti M, Romanazzi I, Romiti GF, Farcomeni A, Lip GYH. Real-world use of Apixaban for stroke prevention in atrial fibrillation: A systematic review and meta-analysis. Stroke. 2018;49: 98–106.
